# Rapidly evolving asymptomatic eosinophilia in a patient with lung adenocarcinoma causes cognitive disturbance and respiratory insufficiency: Case report

**DOI:** 10.3892/ol.2012.1020

**Published:** 2012-11-09

**Authors:** CHENG-HSIANG LO, YEE-MIN JEN, WEN-CHIUAN TSAI, PING-YING CHUNG, WOEI-YAU KAO

**Affiliations:** 1 Departments of Radiation Oncology, Tri-Service General Hospital, National Defense Medical Center, Taiwan, R.O.C; 2 Pathology, Tri-Service General Hospital, National Defense Medical Center, Taiwan, R.O.C; 3 Division of Hematology and Oncology, Department of Internal Medicine, Tri-Service General Hospital, National Defense Medical Center, Taiwan, R.O.C

**Keywords:** lung cancer, eosinophilia, paraneoplastic syndrome

## Abstract

Paraneoplastic eosinophilia is an unusual manifestation that usually remains asymptomatic. In this report, we presented the case of an 82-year-old patient with poorly differentiated lung adenocarcinoma and asymptomatic eosinophilia. The patient’s condition worsened rapidly over a week, with episodes of cognitive disturbance, shortness of breath and acute kidney dysfunction. These symptoms were associated with a 4-fold increase in circulating eosinophil counts. The poor condition hindered further anticancer treatment. Treatment of the eosinophilia with corticosteroids and hydroxyurea significantly reduced circulating eosinophil counts to below the initial levels. Results of this case report suggested that lung cancer patients should be monitored closely for rapidly worsening symptoms of cognitive disturbance and respiratory insufficiency as signs of life-threatening asymptomatic eosinophilia, in order to initiate corticosteroid treatment.

## Introduction

The development of eosinophilia within solid tumors is a rare manifestation, accounting for ∼1% of all cancer patients (1). Since a number of medical conditions are associated with eosinophilia (2), paraneoplastic eosinophilia is diagnosed by exclusion. Depending on the etiology, the consequences of paraneoplastic eosinophilia may range in severity from asymptomatic to life-threatening. Eosinophilia is usually treated successfully with corticosteroids. Paraneoplastic eosinophilia has been reported in a few cases of lung cancer, including lung squamous cell carcinoma (3,4), non-small-cell lung carcinoma (5) and lung adenocarcinoma (6). In the latter case, the patient succumbed rapidly following a tumor relapse associated with rapidly evolving eosinophilia. These studies emphasize the importance of identifying the early signs of aggressive paraneoplastic eosinophilia to initiate corticosteroid treatment prior to end-organ failure.

The transition from asymptomatic to life-threatening paraneoplastic eosinophilia is rapid and difficult to diagnose upon summary examination of the patient, particularly in lung cancer patients who are expected to suffer from respiratory complications. While paraneoplastic eosinophilia is often linked with the overexpression of interleukin (IL)-5 in tumor cells, this type of diagnosis is impractical for such a rapidly evolving and life-threatening complication (4).

In this report, we present a case of paraneoplastic eosinophilia in a patient diagnosed with lung adenocarcinoma. The condition of the 82-year-old male degenerated suddenly, as circulating eosinophil counts increased 4-fold over a few days. The patient experienced cognitive disturbance and shortness of breath, which may represent new diagnostic tools for early corticosteroid treatment to avoid organ damage. The study was approved by the Ethics Committee of the Tri-Service General Hospital, National Defense Medical Center, Taiwan, R.O.C. Informed consent was obtained from the patient’s family.

## Case report

An 82-year-old male was admitted to our hospital on October 5, 2011, with a 2-week history of right-sided flank pain and abdominal fullness. An abdominal sonogram revealed a huge liver mass and the patient was then admitted to our gastrointestinal (GI) section. The patient had a history of well-controlled chronic obstructive pulmonary disease (COPD), hypertension and benign prostate hyperplasia. The patient had herniorrhaphy 1 year earlier and had received amlodipine, tamsulosin and PRN ipratropium/albuterol turbu-haler. The patient had no known allergies and had smoked half a pack of cigarettes per day for 40 years, after which the patient quit for 20 years.

Laboratory data revealed the following: white blood cells, 52,310 cells/*μ*l with 46.3% neutrophils and 45.4% eosinophils; 13.3 g/dl hemoglobin and 242,000 cells/*μ*l platelets; renal functional insufficiency with 36 mg/dl blood urea nitrogen (BUN) and 1.4 mg/dl creatinine; a routine stool test revealed no evidence of parasite infection; immunoglobulin E level was 99.1 IU/ml and the levels of tumor markers in the blood, including carcinoembryonic antigen (CEA; 6.47 ng/ml) and cancer antigen (CA) 19-9 (49.81 U/ml), were elevated.

**Figure 1.**
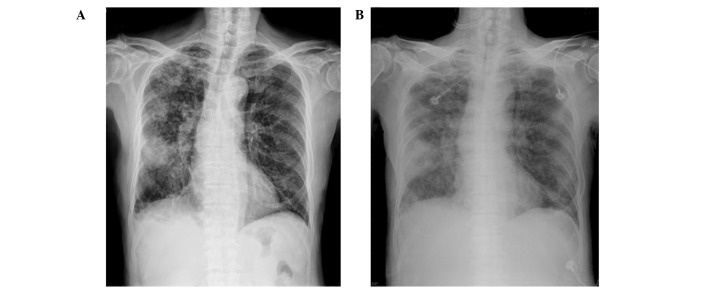
Chest radiograph shows an ill-defined mass lesion ∼5 cm in size at the (A) right middle lung zone at first admission and (B) diffuse infiltration and ground glass opacities over bilateral lung fields outside the mass at readmission.

**Figure 2.**
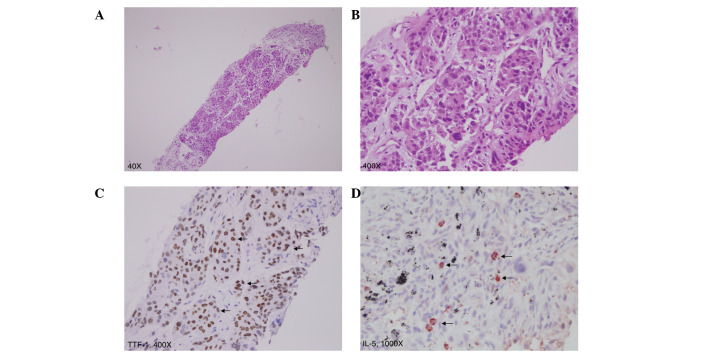
Lung histology showing (A and B) solid nests of tumor cells with nuclear pleomorphism, hyperchromatism and high nuclear/cytoplasmic (N/C) ratios arranged in a solid and focal acinar pattern infiltrating the stroma, which is consistent with poorly differentiated adenocarcinoma. (C) Immunohistochemical staining of lung tumor tissue using thyroid transcription factor-1 (TTF-1) and (D) monoclonal mouse anti-human IL-5 antibody, demonstrating that the tumor is of lung origin and has a specific expression of IL-5 (indicated by arrows).

On admission, crackles were heard in the right lower lung field. Abdominal palpation revealed mild epigastric tenderness without muscle guarding. A chest radiograph revealed an ill-defined mass lesion ∼5 cm in size in the right middle lung zone (Fig. 1A). Computed tomography of the chest revealed a right middle lung lobe mass and multiple variable-sized nodules in the two lung fields. Computed tomography of the abdomen demonstrated several peripherally enhancing lesions in the lobes of the liver. Magnetic resonance imaging of the brain revealed no evidence of metastasis. Whole-body bone scan revealed multiple bone metastases. Biopsies of the liver and lung mass were performed and pathology revealed poorly differentiated adenocarcinoma of the lung, positive for thyroid transcription factor-1 (TTF-1; Fig. 2A–C). Definitive oral-targeted therapy was advised if epidermal growth factor receptor (EGFR) abnormality was present due to the patient’s end stage and old age.

**Figure 3.**
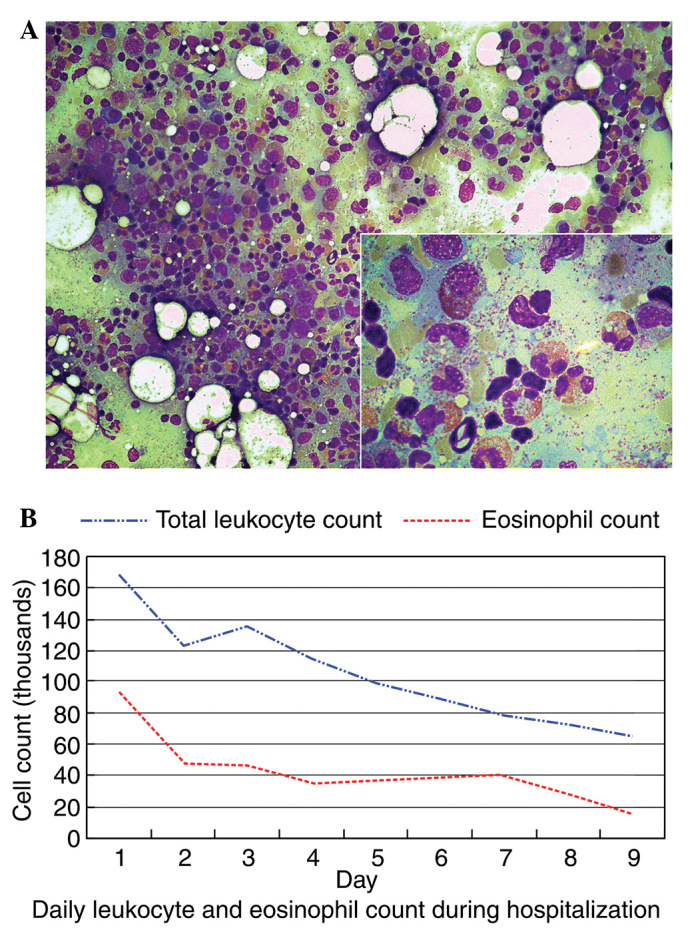
(A) Reactive bone marrow hypercellularity with high eosinophil counts, which has an average percentage of >30%, compared to 1–5% in normal bone marrow. Inset: high-power field shows scattered normal eosinophils. (B) Daily total leukocyte and absolute eosinophil counts were reduced progressively with the combination treatment of hydroxyurea and steroid.

The patient was discharged following completion of the staging work-up and waited for the result of the EGFR analysis. One week later, the patient was readmitted for cognitive disturbance and shortness of breath. On arrival, the patient was noted to be agitated and disoriented and had disorganized speech. Physical examination revealed diffuse wheezing over all lung fields. Pitting edema was noted on the legs. The peripheral white blood cell count had increased 4-fold over a week (168,800 cells/ml), with a proportional increase in eosinophil counts (55.2%). Elevated potassium (5.6 mmol/l), uric acid (13.8 mg/dl), creatinine (2.7 mg/dl) and lactate dehydrogenase (LDH; 420 U/l) levels were also noted. Chest radiography demonstrated diffuse infiltration and ground glass opacities over the two lung fields in addition to the previous finding (Fig. 1B). Brain computed tomography presented no special findings. Bone marrow biopsy was performed, which revealed reactive bone marrow hypercellularity with a markedly high eosinophil count (Fig. 3A). The average percentage of eosinophils was 39%, compared to 1–5% in normal bone marrow. Chromosomal analysis demonstrated normal karyotype. Immunohistochemical analysis using the monoclonal mouse anti-human IL-5 antibody (R&D Systems, Minneapolis, MN, USA) demonstrated that IL-5 was specifically expressed in tumor cells (Fig. 2D). Lung cancer-associated paraneoplastic eosinophilia and acute renal dysfunction were diagnosed.

The patient was treated with hydration and allopurinol to control extreme hyperuricemia. Due to the old age and weak condition of the patient, anticancer treatment was not provided. Since eosinophilia-related organ damage was suspected, hydroxyurea and corticosteroid were administered to reduce the number of eosinophils. The white blood cell count was reduced significantly after 9 days of treatment to 65,500 cells/*μ*l with 23% eosinophils (Fig. 3B). The patient’s conscious state, kidney function and blood cell count improved following treatment. However, the dyspnea persisted and the patient acquired pneumonia 4 days after the second admission. The family refused further treatment and intervention due to the poor prognosis. The patient succumbed to healthcare-acquired pneumonia with severe sepsis due to *Pseudomonas aeruginosa,* 10 days after admission.

## Discussion

The present report describes a case of lung adenocarcinoma complicated by severe and aggressive eosinophilia. A number of medical conditions, including allergic disorders, parasitic and fungal infections, vasculitis and drug reactions, as well as hematologic and non-hematologic malignancies are associated with eosinophilia (2). The fact that our patient did not present any of these conditions supports the paraneoplastic nature of the eosinophilia. The pathogenesis of paraneoplastic eosinophilia is unclear. Numerous mechanisms have been postulated and bone marrow stimulation by cytokines secreted by tumor tissues, including granulocyte macrophage-colony stimulating factor (GM-CSF), G-CSF, IL-3 and IL-5, is most commonly reported (4,7–11). In our case, the immunoreactivity of tumor cells to IL-5 is consistent with that reported in these previous studies.

Patients with paraneoplastic eosinophilia are typically asymptomatic. However, in a number of cases, a markedly elevated eosinophil count may be associated with shortness of breath and wheezing. In the present case, the patient exhibited shortness of breath and cognitive disturbance in the form of agitation, disorientation and disorganized speech. Normally, anticancer therapies also resolve the eosinophilia. Matsumoto *et al* reported a return to normal hematologic status with chemotherapy (12) and Pandit *et al* demonstrated that leukocytosis and eosinophilia normalize following tumor removal (4).

Primary eosinophilic syndromes are managed successfully with corticosteroid therapy (13–15). However, a number of patients are non-responsive to corticosteroids, but respond well to hydroxyurea (16). Hydroxyurea is also reported to be an effective first-line agent in hypereosinophilic syndrome (15). A combination of hydroxyurea and corticosteroid increases the response rate (15). However, there is no standard treatment for paraneoplastic eosinophilia. To prevent potential harmful effects from chronic exposure of organs to excessive eosinophils, we used a combination of corticosteroid and hydroxyurea, which led to a marked improvement in blood cell counts. The significant effect of corticosteroid and hydroxyurea in reducing the eosinophil count may play a role in improving and stabilizing paraneoplastic eosinophilia and act as a bridge to more anticancer therapies.

The clinical significance of eosinophilia in cancer patients is undefined. Iwasaki *et al* report that tumor-associated eosinophilia is associated with a good prognosis (17). However, more studies support the view that paraneoplastic eosinophilia reflects a more extensive disease and poor prognosis (7,18–21). Anagnostopoulos *et al* suggested that the return of eosinophilia may be an indicator of tumor recurrence (10). In our case, the extremely high eosinophil count and its rapid rise suggested aggressive disease progression and poor prognosis. The addition of combination therapies (corticosteroid and hydroxyurea) to anticancer drugs in paraneoplastic eosinophilia may be beneficial to patient prognosis.

In conclusion, this is the first report of cognitive impairment in combination with respiratory insufficiency as symptoms of rapidly worsening paraneoplastic eosinophilia (eosinophil surge) in cancer patients. This condition may be used for an early diagnosis to initiate corticosteroid treatments and avoid organ damage. This case also suggests that lung cancer patients who present abnormally high counts of eosinophils, should receive a combination of corticosteroids, hydroxyurea and anticancer drugs to prevent the development of aggressive and life-threatening eosinophilia, even if they are asymptomatic initially. This is likely to also enhance the benefits of the anticancer treatment.
